# Efferent Copy and Corollary Discharge Motor Control Behavior Associated with a Hopping Activity

**DOI:** 10.4172/2165-7025.1000167

**Published:** 2013-07-23

**Authors:** Wangdo Kim, António P Veloso, Filipa João, Sean S Kohles

**Affiliations:** 1 Univ Tecn Lisboa, Fac Motricidade Humana, CIPER, LBMF, Estrada da Costa, P-1499-002 Lisbon, Portugal; 2 Regenerative Bioengineering Laboratory, Departments of Mechanical & Materials Engineering and Biology, Portland State University, Portland, Oregon, USA

**Keywords:** Synaptic enhancement, Gibson’s affordances, Muscle springs, Afferent, Reafferent, Homeostasis, Bernstein’s DOF reduction

## Abstract

Hoppers respond not only to stimuli from the ground surfaces but also to cues generated by their own behaviors. This leads to desensitization because although the afferent and reafferent signals have distinct causes, they are carried by the same sensory channels. From a behavioral viewpoint, it may be necessary to distinguish between signals from the two causes especially when monitoring changes in the external environment separate from those due to self-movement. We were able to separate afferent sensory stimuli from self-generated, reafferent signals using an action-oriented perception system and dynamic programming approach. This effort addressed the question of how the nerve system selects which particular degree of freedom (DOF) to cancel reafferent input. We have proposed an internal one-DOF model characterizing the motor control system during hopping, allowing the generation of an estimated ground reaction signal to drive natural shock absorption of the leg.

## Introduction

The French physiologist Claude Bernard first described the difference between an organism’s internal environment and its external environment [1]. Bernard concluded that the internal environment served as a kind of buffer between living cells and the fluctuating external environment. Later, American researcher Walter Cannon revised Bernard’s suggestive ideas into a much more detailed form. In his popular book, The Wisdom of the Body [2], Cannon coined the term homeostasis for the modern concept of biological self-regulation. Cannon’s idea became a central concept in physiology, later borrowed by scientists in several other fields as well.

The problem described herein is a popular formulation of biomechanical regulation where by the motivation is to keep the state of the system close to equilibrium [3]. Such problems are common in the theory of optimal control of a motion or a process. The basic task of a control system is to manage the relationship between sensory variables and motor variables. There are two basic kinds of transformations that can be considered: sensory-to-motor transformation and motor-to-sensory transformation. The transformation from motor variables to sensory variables is accomplished through the environment and musculoskeletal systems; these physical systems transform efferent motor actions into reafferent sensory feedback [4,5]. While the notion goes back to Von Helmholtz [6], the modern concept of “corollary discharge” and “efference or efferent copy” was put forward previously [7].

### Action-oriented perception system

Optimization theory provides a computational framework which is natural for a selection process such as motor planning [4]. In the optimal control approach, movement trajectories are not explicitly planned but are a consequence of the objective function and the system’s dynamics. The problem, however, of coordinating the different muscle groups involved in the repetitive sequence of skeletal activities, such as locomotion, has not been given sufficient attention from the perspective of control theory. In fact, the musculoskeletal system with all of its physiologic actuators represents a large control system. Therefore it is interesting to explore a functional characterization obtained by using a systematic approach to muscle control.

Model-based representation control strategies are those that rely on accurate internal models of the environment. These are constructed from a combination of perceptual information and prior knowledge. However, prior knowledge represents the primary source information for planning and executing actions even in the absence of perceptual information [8]. Forward models are a predictive internal approach for motor control that takes the available perceptual information, combined with a particular motor program including dynamic optimization [9] or static optimization [10], and tries to predict the outcome of the planned motor movement [11].

Most work in this vein of thinking has focused on the nature of the objective function, such as minimizing energy, motor variance, or performance errors. This approach requires complete information in the control algorithm about the controlled objects. In this application it would be necessary to account for the underlying physiological properties of muscle in order to obtain more accurate estimates of muscle force. An alternative to model-based control is that of information-based control. Informational control strategies organize movements and action based on perceptual information of the environment, rather than on cognitive models or representation of the external world. The actions of the motor systems are organized by environmental information and information about the current state of the functioning agent [12]. A core assumption of information-based control strategies is that perceptions of the environment are rich in information and veridical for the purpose of producing actions. This approach runs counter to the assumption of indirect perception made by model-based control strategies.

**Figure 1:**
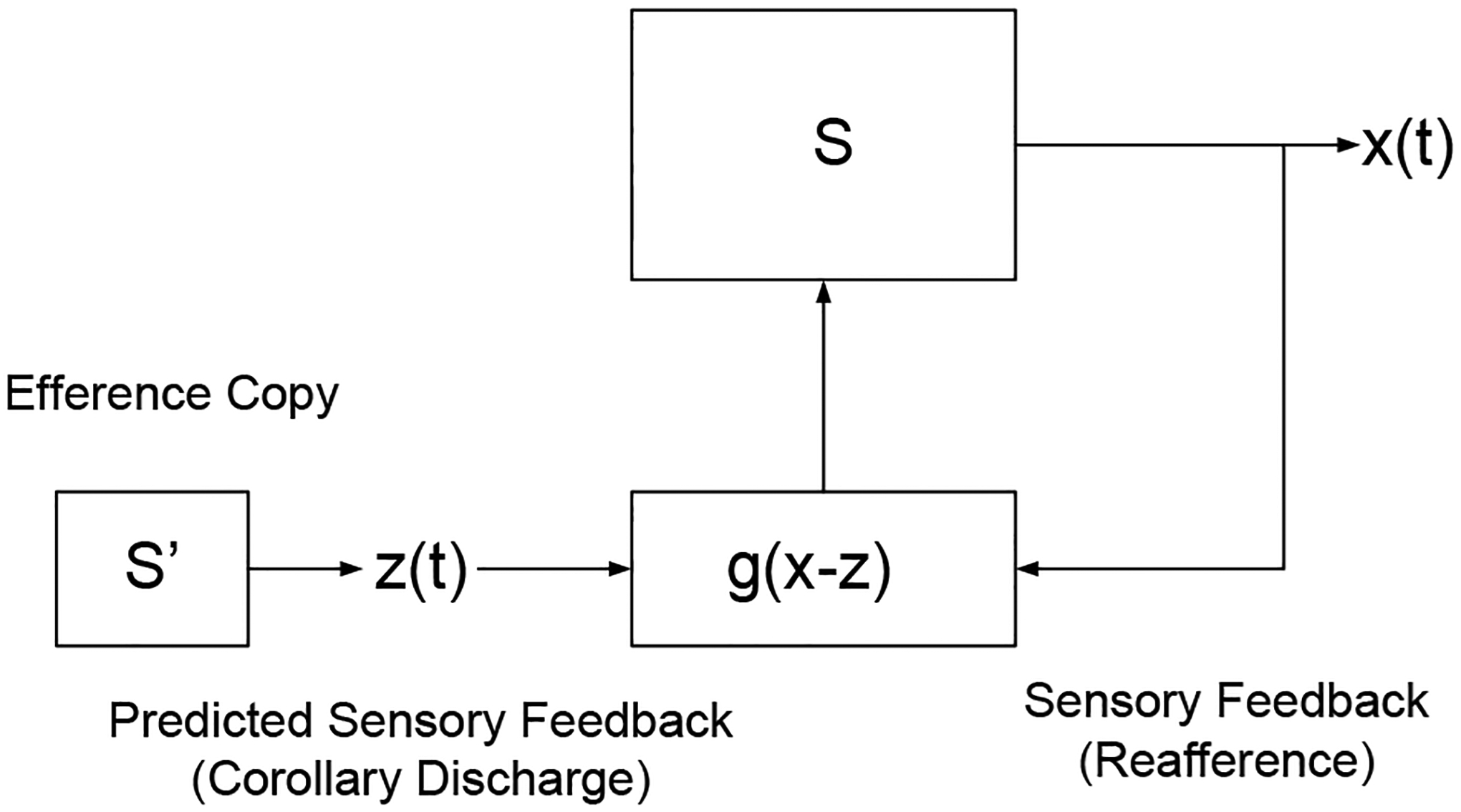
A diagram of the feedback loop whereby the very deviation of the system from its desired performance is a restoring force to guide the system back to its proper functioning. An efference copy is used to generate the predicted sensory feedback (corollary discharge), which estimates the sensory consequences of a motor command. The actual sensory consequences of that motor command are compared with the corollary discharge to inform the central nervous system (CNS) about the external actions. Schematically, this is shown here as the actual leg spring system S with the state x(t), while S’ is another hypothetical system with state z(t). The “driving action” z(t) is supplied as the input to the system S, representing the instruction of what will be the output variable x for the object. These two system, x(t) and z(t), are compared, resulting in a resulting forcing influence g(z-x), which is then exerted upon S.

In this study, we consider an information-based control approach with partial information about the objects and active information storage in the control process. Central to the information-based approach will be observed experimental sensory data. Suppose that we observe an output function x in the time interval t, where a ‘black box’ manipulation is comprised of subsystems with each system subject to differential equations in which unknown parameters of, or are generally, of unknown types. It is desired to determine the nature of the black box and, if we have control over the system, this may be regarded as an adaptive feedback problem, in which its gathered information gives an indication for what input will now provide the most helpful additional information (Figure 1). The objectives here are similar to a previously prescribed dual control theory [13], which suggests that adaptation can become specifically tuned to identify task-specific parameters in an optimal manner.

## Model Development

The motor behavior of each subject’s hopping activity is analyzed in terms of an action-perception cycle rather than stimulus and response. The hopping individual uses an internal model of the world to predict, not infallibly, the results of interacting with the surface in optimal policy. In this section, we establish the way in which action and perception are integrated, whether in intelligent human behavior, the activity of animal, or the smooth functioning of an adapted robot.

**Figure 2:**
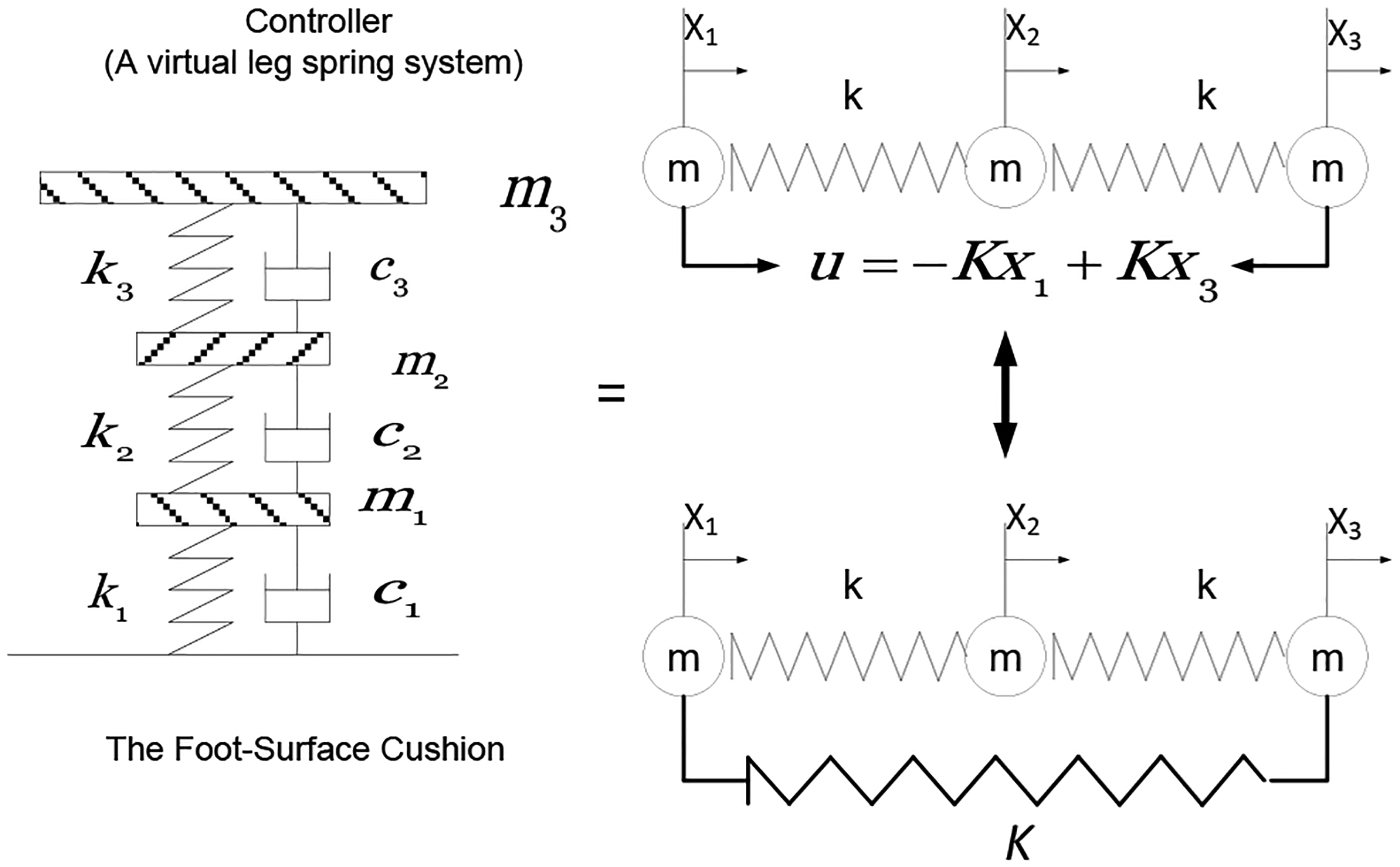
The three DOF leg spring model demonstrates how the system can perform the vibration suppression function. The model consists of SDOF of the foot-ground contact and the attached MDOF of the dynamic controller (two degrees). The equivalent active and passive system is illustrated (with parameters listed in Table 1). The control force simply adds the stiffness to the system. In other words, the active control system with the control input is equivalent to the passive system with springs of stiffness k_i_.

We applied the feedback control approach (Figure 1) to a three DOF leg spring chain in a position of stable equilibrium under the influence of leg stiffness (Figure 2 and Table 1). If the leg spring receives a displacement during hopping, the forces will no longer equilibrate, but the system will be exposed to the action of a force on a leg spring chain. The basic idea is to use the very deviation of the system from its desired performance as a restoring muscle force to guide the leg spring back to the proper functioning.

The performer will commence to move with an optimal policy when the central nervous system (CNS) is permitted to select its own forward model from the available modes defining the freedom of action. A forward model uses the efference copy [7] to anticipate and cancel the sensory effects of movement (Figure 1). Sensory signals arise in the periphery due to two causes: those resulting from environmental disturbances on the body (afference) and those resulting from self-generated movement (reafference) as the sensory consequences of the movement itself [14]. From a behavioral viewpoint, it may be necessary to distinguish between signals from the two causes especially to monitor changes in the external world separate from those resulting from self-movement. The internal sensory signals need to cancel reafference has been labeled corollary discharges [7]. This approach demands constant perception of the system. Alternatively, an additional extraneous force y(t), determined a priori as a known function of time, can be applied to the system in such a way as to keep x(t) as close as possible to z(t). This provides an alternate concept that is a model-based approach to determine the trajectories without further reference to the behavior of the subject.

### The Single DOF Internal Model and Policy

In this study, we assume that the nervous system must have its own internal model as a simple representation of the body’s dynamic interaction with the surrounding environment. When observing a hopping individual, they learn the movement first by reducing their DOFs through muscle stiffening in order to have focused control over the activity, they then tune themselves to the surface, gradually “loosening up”, exploring the available DOFs as the task becomes more comfortable, and from there find an optimal repeated motion similar to a natural vibration mode.

**Figure 3:**
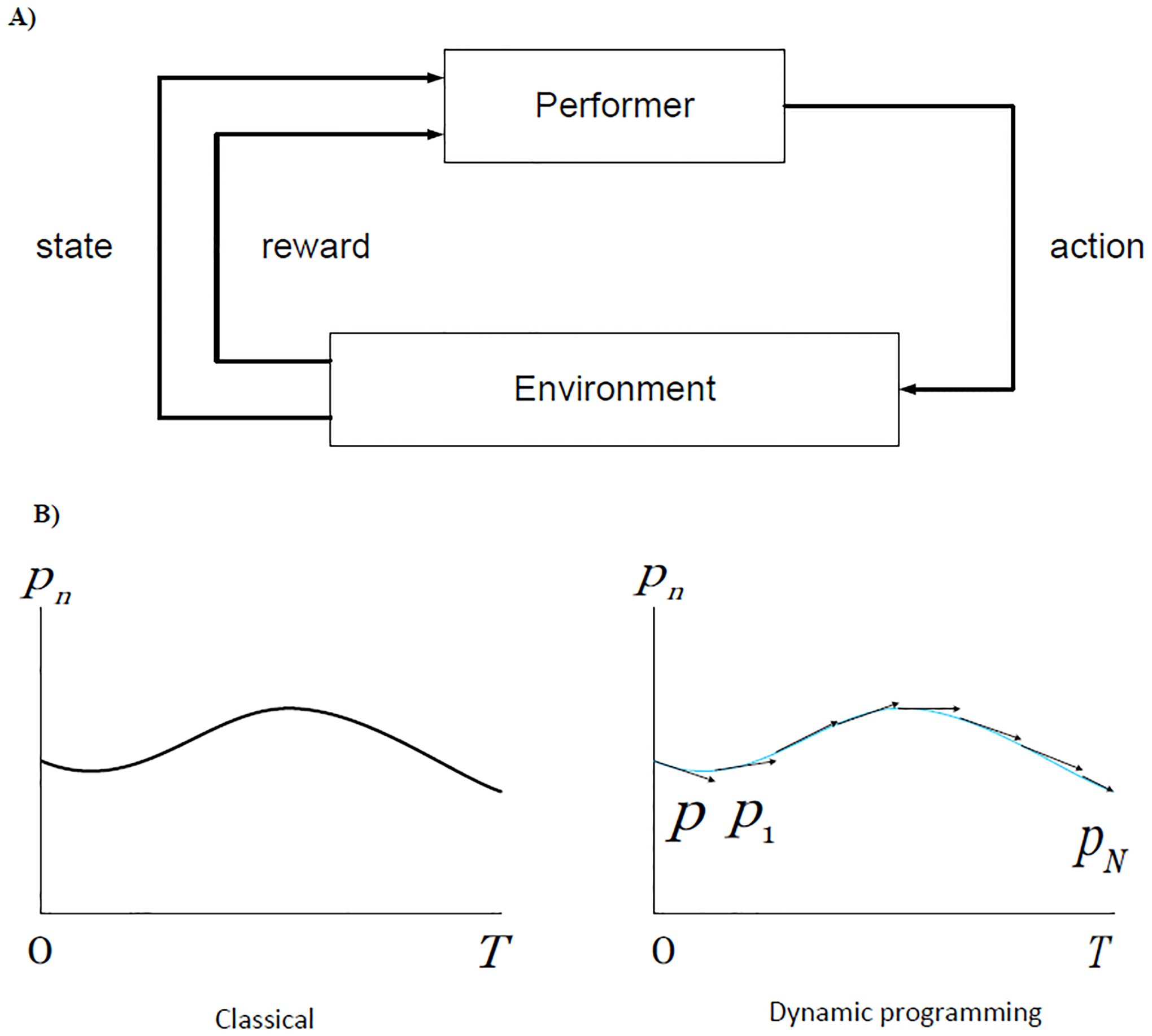
(A) The performer-environment interaction schematized as an adaptive control process indicating the interactive influences of the state, optimized rewards, and action. (B) Classically one seeks a curve *p* defined over the given interval [*o*,*T*] which maximizes. In our approach, at each point, we seek a direction which is optimal; the solution is obtained in the form of a policy, a set of instructions for carrying out the process.

As introduced, the dynamics of a leg spring may be simplified with three DOFs consisting of three interconnected masses with three springs (Figure 2). The equivalence between an active motor synergy and a passive system in this spring model has been previously verified and illustrated [15]. The problem we wish to consider here is how a performer gradually adapts his/her behaviour to the information that he/she receives in an environment possessing unknown features during hopping. First, approximation in policy space leads to quasilinearization to define the influencing parameters. The policy depends on the particular choice of harmonic modes representing the activity. Dynamic programming then determines the exact control forces to match the desired ground reaction forces (GRFs). Previous research has described a computational technique for the determination of interaction parameters based on the observation of interacting performers with their environment [15–20]. Human movement control can be seen as a process that is distributed systemically over the performer-environment system, rather than being localized within an internal structure associated with the performer [21]. The learner and decision-maker are identified as the performer. The objects the performer interacts with, comprising everything external to the performer, is called the environment (Figure 3). The environment also gives rise to rewards, special functions or values that the performer tries to maximize over time. During hopping, the reward is to suppress vibration as a functional or natural shock absorber [15]. We have postulated that rather than having the motor control be localized either as an internal model of the performer or between his/her environment (for example, the foot-surface interaction), control of the shock absorption is distributed over the performer-surface system. Thus, we predict that the performers respond not only to stimuli generated as a by-product of the performer’s own behavior, but also to the environment, or afferent, sensory stimuli. Such self-generated, or reafferent, sensory information, is used to update or fine-tune the ongoing motor act [22] and, in active sensory systems, to help monitor the environment [23]. Movements and postures are controlled and coordinated to realize functionally specific acts that are themselves based on the perception of affordances, i.e., possibilities for actions [22]. More specifically, the performer and environment interact at each sequence of discrete time steps, t=0, 1, 2, 3… At each time step t, the performer receives some representation of the environment’s state and on that basis selects on action. Sensory signals arise in limb periphery from two causes: those as results of environmental influences on the body, and those resulting from self-generated movement. The first are termed afference, while the second types of sensory signals are known as reafference as they are the sensory consequences of movement. Although the afferent and reafferent signals have distinct causes, they are carried by the same sensory channels. From a behavioral viewpoint it may be necessary to distinguish between signals from the two causes especially when monitoring changes in the external world separate from those resulting from self-movement. An analogy can be drawn between the control function and an individual who interacts with their environment. The individual studies their surroundings in order to influence them in a useful manner. But in order to direct their actions better, they must understand the environment better; therefore the individual may act on the environment not in order to obtain a direct advantage, but with the aim of improving their environmental understanding. Thus the influence on the environment and the study of it are closely linked.

We propose an information based control theory whereby human locomotion is neither triggered nor commanded, but controlled. The basis for this control is the information derived from perceiving oneself in the world. Control therefore lies in the human-environment system. Human movement control can be seen as a process that is distributed over the performer-environment system, i.e., rather than being localized in an internal structure within the performer [22]. The performer and his/her environment (reaction surface) may be said to be co-participants in any resulting action. In this way, actions are specific to function rather than to mechanism [24]. Movements and postures are controlled and coordinated to realize functionally specific acts that are themselves based on the perception of affordances, i.e., possibilities for actions [21]:

“The rules that govern behavior are not like laws enforced by an authority or decision made by a commander, behavior is regular without being regulated. The question is how this can be (p. 225).”

Although the statement asserts Gibson’s belief that behavior is regular without being centrally controlled, the question of how this exists remains unanswered. Motor behavior may be viewed as a problem of maximizing the utility of movement outcome in the face of sensory, motor, and task uncertainty [25]. Viewed in this way, Bellman pointed out (having previously developed dynamic programming) that in order to understand how an organism gradually adapts its behavior to the information that it received in an environment possessing unknown features, there is the issue that learning and performing occurs simultaneously [26]. The probabilistic aspect of this issue is not a “strange” element or an addition to the basic “regular” theory. The issue introduces constraints into the structure of automatic control theory, being an essential part while not explicitly addressed in this study.

The conclusion to which we are slowly wending our way is that a new genre of mathematical problem has arisen in the last few years, that of controlling a large system, here the musculoskeletal system. The human skeleton represents a mechanical linkage system with many degrees of freedom (DOFs); furthermore, we do not address the ambitious question of control rather we introduce a feasible operation theory [27].

In this study we consider a vertical hopping activity as an application of the control theory within a large self-regulating biological system. This application is a special case of action-oriented perception control of human movement and can be addressed via the dynamic programming algorithm. Proponents of direct perception [21] suggest that the relevant information encoded in sensory signals is not derived from the physical properties of interacting objects, but rather the action potentials the environment affords. These affordances are directly perceivable without ambiguities, and preclude the need for internal models (states) of representations of the world.

An irony of science is that in order to understand complexity, we must often throw away information. In order to reduce a complex system to its simplest form; we have introduce an approach based on a single DOF. Discovering the basic principles that underlie the reduction of DOFs is one of the major challenges understanding the motor control of limb movement. We bring forward a hypothesis, proposing that the reduction of the number of DOFs serves a direct perceptual purpose. The general aim of this study is therefore to indicate that the perception of possibilities for action, i.e., affordances, may be represented as action potentials associated with how the nerve system selects which particular DOF to use.

We apply a forward modeling approach for the single DOF because we assume its action potential is directly related to the concept of affordance. An important new “policy” is embedded in a performer’s inter-model in that a single DOF can execute no movement, which is not a motion about one definite mode. The purpose of this paper is therefore to identify the forward model for a hopping activity that uses a copy of the motor command, an “efference copy”, to anticipate and cancel the sensory effects of the movement, the “reafference.” We therefore also show that in the case of limb control during hopping, the efference has an additional sub-function of canceling the effects on sensation induced by self-motion and distinguishing self-produced motion from the sensory feedback caused by disturbances with objects in the environment.

## Materials and Methods

### Multistage decision process

We shall consider the use of dynamic programming (DP) as a computational algorithm capable of yielding numerical answers to a multistage decision process. Bellman recognized that many biological systems display a number of characteristic in motor behavior similar to a decision process [25]. He proposed an optimization policy having the property that whatever the initial state and initial decision are, the remaining decisions must be optimized with regard to the state resulting from the first decision. The term dynamic programming refers to a collection of algorithms that can be used to compute optimal policies given a perfect model of the environment via a Markov decision process (MDP). Thus, DP opens up a whole new class of decision-making solutions and has paved the way for modern control theory approaches.

Having introduced a mathematical definition of a system, let us now precisely define the intuitive concept of process. Analytically, we conceive of a system as a state vector *x*(*t*) and a rule for determining its value at any time *t*. We shall replace a symbol *x*(*t*) by the symbol *p*, and think of *p* as a point in a set or space *R*. Next we consider a function *T*(*p*) as a transformation with the property that the transformed point *p*_1_ = *T*(*p*) belongs to *R* for all *p* in *R*. Intuitively, *p* represents the initial state of a system and defines *p*_1_ = *T*(*p*) as the state one time unit later. Generally, the set of vectors are established as:

(1) $$\left[p,p_{1},p_{2},\ldots ,p_{n},\ldots \right],$$

Where *p*_0_=*p* and *p*_*n*+1_ = *T*(*pn*), *n* = 0, 1, 2, …, represents the time history of a system observed at the discrete times *n* = 0,1,2,…, the successive states of the system.

Let us begin with a generalized version of the control process. Let *p* be a point in a posed space *S* specifying the state of a system and *q* be a point in decision space *D*. To define a multistage decision process of the simplest type, we start with the previously established notion of a multistage process *p*_1_ = *T*(*p*). We now enlarge this concept by taking the transformation *T* to depend on another vector as well, *T* = *T*(*p,q*). If the decision equivalent to *q* is made when in state *p*, then the system is transformed into the state defining p′ as:

(2) $$p^{\prime }=T(p,q),$$

In this application, we wish to concentrate upon policies having the simpler form of:

(3) $$q_{k}=q_{k}\left(p_{k}\right),$$

As a function of the current state and stage of the process.

A return function of *g*(*p*,*q*) is then produced. Employing the Principle of Optimality, we see that the problem of obtaining the maximum total return for an unbound process leads to the functional equation for N iterations:

(4) $$f_{N}(p)=\underset{q}{\text{max}}\left\{g(p,q)+f\left[T(p,q)\right]\right\}.$$

Let then the maximum value of the return function, dependent only upon the initial state *p* and the number of stage *N*, be denoted by *f*_*N*_(*p*). In other words,

*f*_*N*_(*p*)=the total *N*-stage return obtained starting in state *p* using an optimal policy. In geometric parlance, we can say that the classical view is that of a curve as a locus of points, while dynamic programming considers a curve to be an envelope of tangents (Figure 3).

Dynamic programming has the potential for dealing with problems of control and sequential decision making under uncertainty [28]. In this theoretical approach, we possess the tool now defining ‘approximation in policy space’.

To study the structure of the solution associated with the foregoing equations, we can employ various kinds of successive approximation. We can approximate the solution of the functional equation *f*(*p*), or we can approximate to the optimal policy function *q*(*p*). The important point here is that Equation (4) involves two functions: *f*(*p*), the return function and *q*(*p*), the optimal policy function.

So far we have assumed that the result of the transformation *T* is to take the state vector *p* into the set of vectors *p* and then into the state vector *p*_1_, where *p*_1_ is uniquely determined by *p* with perfect state information. In this study, however, since the parameters are unknown, T is not completely known. This means that a stochastic process must replace the deterministic models we have been using. In place of the statement that *p*_1_ is unknown, we suppose that *T* is a stochastic transformation, which produces a random vector *p*_1_ whose probabilistic distribution is determined by *p*. To illustrate this idea, let us begin with the three unknown spring constants *k* where *p*_*k*_ is determined by the relation:

(5) $$p_{n}=T\left(p_{n-1},k_{n}\right),n=1.2,\ldots$$

Where *p*_0_ = *p* and the *k*_*n*_ is independent random variable with the identical probability distribution *dG*(*k*) and:

(6) $$f_{N}(p)=\underset{k}{\text{exp}}\left[g(p)+f_{N-1}(T(p,k))\right]\;=g(p)+\int f_{N-1}(T(p,k))dG(k),$$

with *f*_*0*_(*p*) = *g*(*p*) Here the notation “exp,” indicates that the expected value is to be taken with respect to the random variables k.

### Applied biomechanics

**Figure 4:**
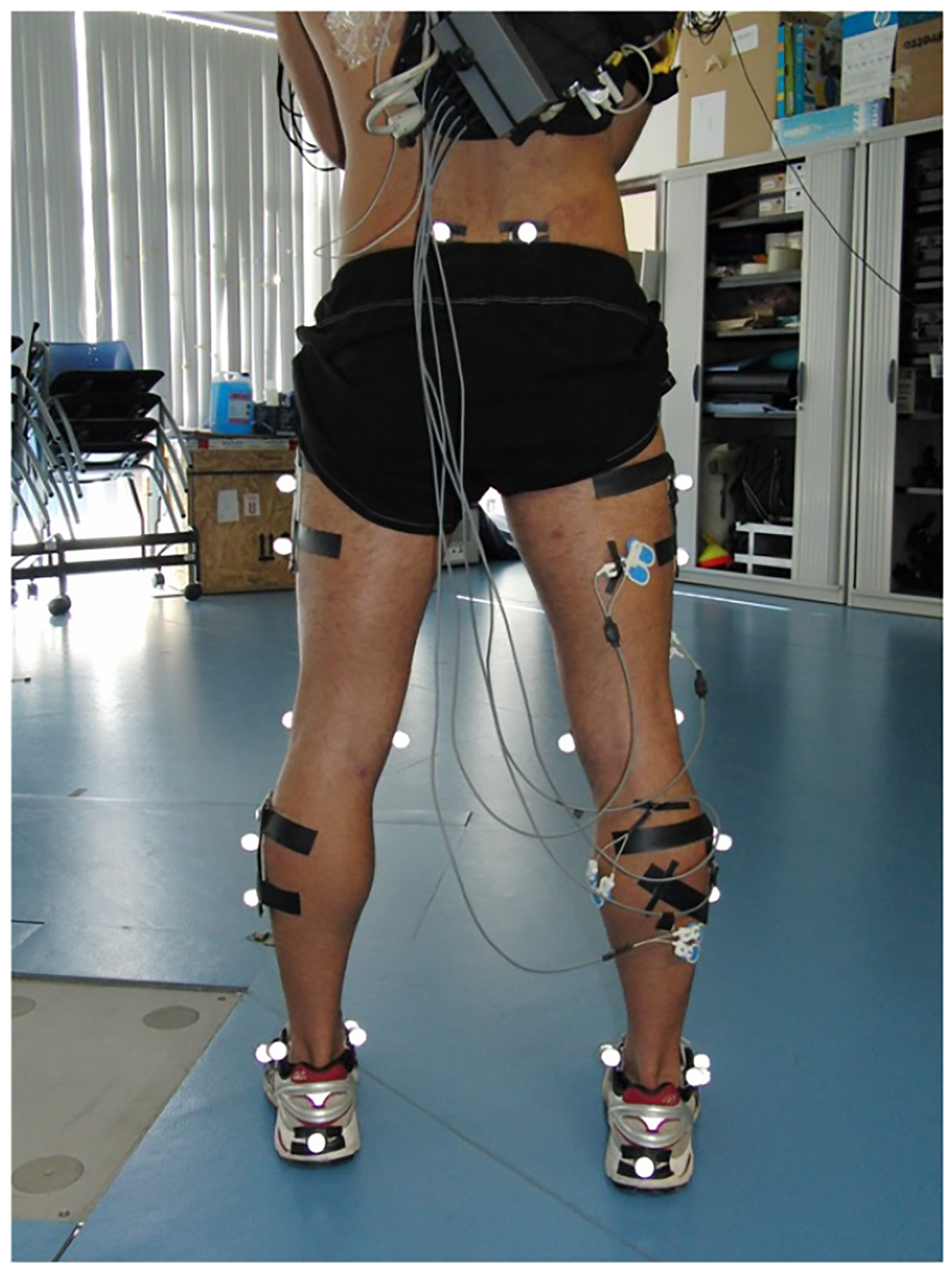
Surface markers (indicated by bright dots) were used to predict the contribution of the individual components of the leg spring during the stance phase of the hop. These markers were used for the reconstruction of six body segments (right and left thigh, right and left shanks and right and left feet). The current image represents the stationary configuration.

Eleven healthy, well-trained subjects (4 women and 7 men) gave their written informed consent to participate in this study (via Institutional Review Board approval). All the subjects performed a sequence of unilateral hops on her/his dominant lower limb until voluntary exhaustion. To determine the 60% peak hop height as a control parameter for the hopping activity, each subject performed a squat jump (SQJ) before and after the hopping routine. The minimum height for each hop during the fatigue regime was set at 60% of the maximum height achieved in the first SQJ. Motion capture was collected with an optoelectronic system of ten cameras (Oqus-300, Qualisys AB, Sweden) operating at 200 Hz. Three vertical displacements at the center of mass of each segment (thigh, shank, and foot) were also processed (Visual 3D, C-Motion, Inc., Canada). The trajectories at the centers of mass of each segment were collected (Figure 4 indicates the surface marker location information).

The electromyographic (EMG) activities of the tibial is anterior (TA), gastrocnemius medial is (GM), soleus (SOL), vastus lateralis (VL), and the biceps femor is (BF) were recorded (Figure 4). The surface electrodes (Ambu Blue Sensor N-00-S/25) were placed with an inter-electrode distance of 20 mm, in accordance with the SENIAM project recommendations [29] and placed as follows. TA: electrodes placed at 1/3 on the line between the tip of the fibula and the tip of the medial malleolus. GM: electrodes placed on the most prominent bulge of the muscle in the longitudinal direction of the leg. SOL: electrodes placed at 2/3 of the line between the medial condyle of the femur to the medial malleolus. VL: electrodes placed at 2/3 on the line from the anterior spina iliaca superior to the lateral side of the patella. BF: electrodes placed at 50% of the line between the ischial tuberosity and the lateral epicondyle of the tibia, in the direction of the line between the ischial tuberosity and the lateral epicondyle of the tibia. A ground electrode was placed over the C7 vertebrae. The EMG data were transmitted by telemetry (Biotel 88, Glonner, Germany) and collected at 1 KHz. The EMG signals were low-pass filtered using a fourth order Butterworth filter with a cut-off frequency of 6 Hz and normalized to peak activity recorded during the hop exercise. The EMG signal was first band passed with a 30 Hz high-pass and a 500 Hz low-pass filter. The EMG signals were then rectified and low-pass filtered at 6 Hz to build the linear envelope [30]. The RMS amplitude was calculated within a window of 125 milliseconds after the liner envelope. Statistical comparisons within and between subjects were made with commercial software (PASW Statistics v18, SPSS, USA).

## Results

**Figure 5:**
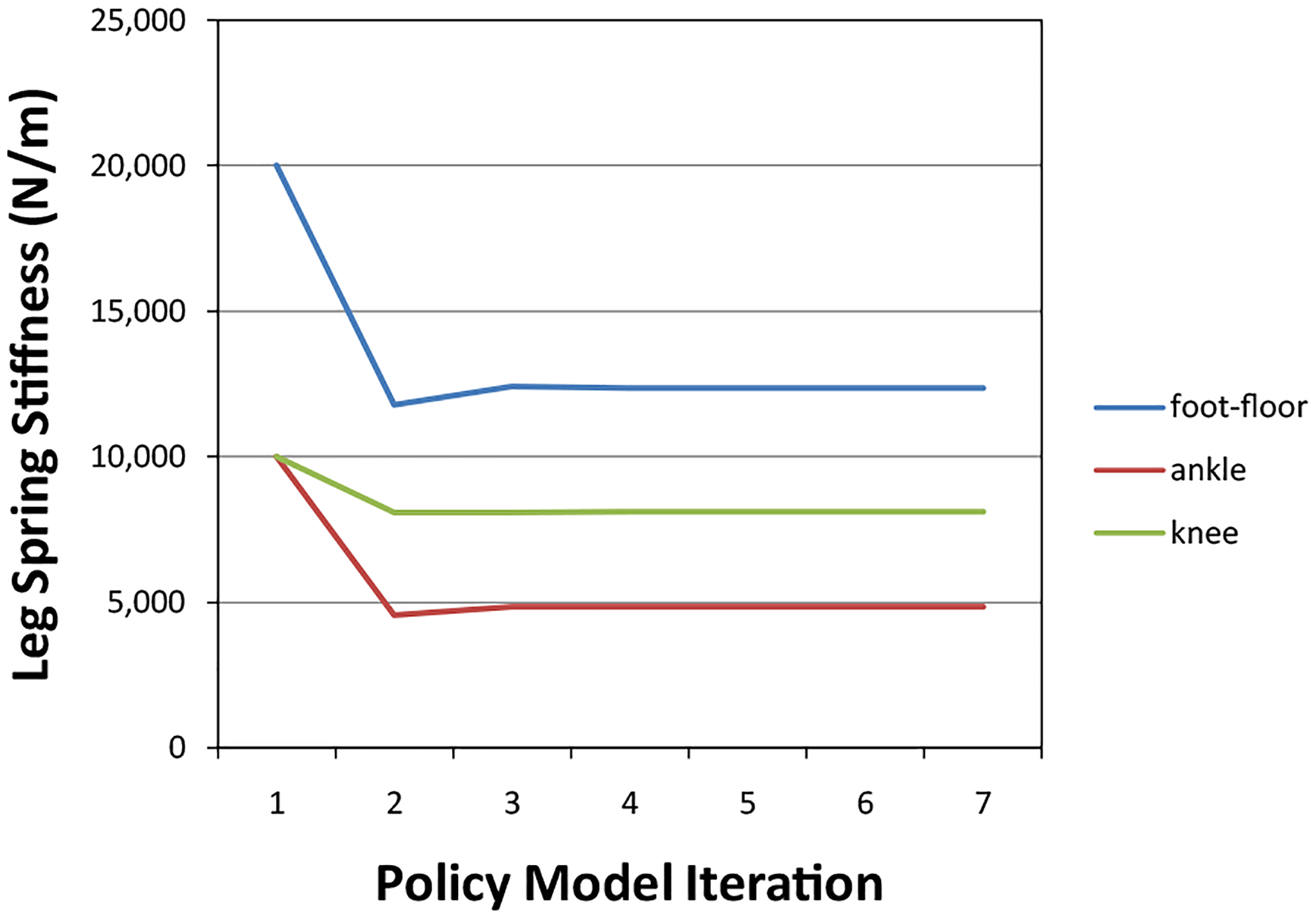
Convergence of leg segment stiffness parameters as determined for a representative subject.

The leg stiffness parameters were found for a representative subject (001-F), showing a fast convergent rate, such that three iteration steps are sufficient to take parameters to converged values (Figure 5). The rule of the policy-iteration method is quite simple, such that the optimal policy has been reached when the policies on two successive iterations are identical.

**Figure 6:**
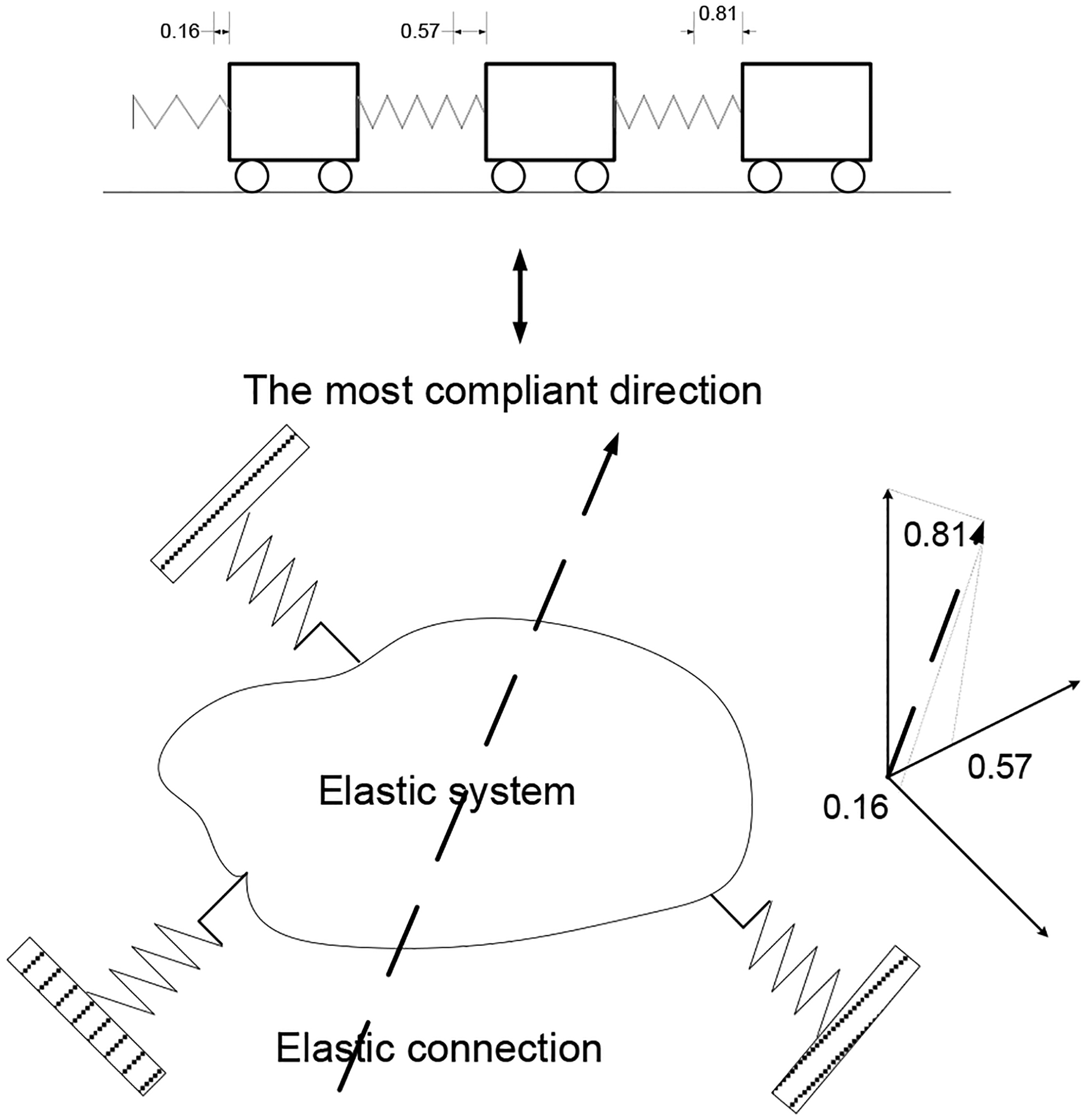
Mode shape schematics and plot for first vibrational mode (6.5 rad/sec), where all masses move in phase with less stress within the connecting springs (tissues). Specific deflection ratios of 0.16, 0.57, and 0.81 are shown in a three-dimensional space with Cartesian coordinates.

A model analysis was then applied to the leg spring model. The modal analysis allows us to reconstruct the overall response of the 3 DOF leg spring within the stance phase as a superposition of the response of three single DOF modes of the system. As an elastic system, leg springs vibrate with the three distinct frequencies (Figure 6). Thus, we postulate that the CNS can select the slowest component and we may assume that to represent the efference copy of the inter model from the available DOFs.

The resulting mode shapes and vibrational modes can be compared with known physiological processes to identify the efference copy mode regimes, which we assume to be the first mode (6.5 rad/sec) that contributed 97.6% of total motion of the leg spring. High frequency modes that have little contribution to the system dynamics can be eliminated, here the second and third modes. We may build a “reduced” model where only the most significant mode, i.e., the first mode is retained. It must be observed that the first mode alone can reasonably represent the mechanics of hopping that is invariant over hopping frequencies [15].

The selected single DOF model, assuming an internal model, has another distinct feature. Consider an elastically suspended leg spring model (Figure 6). If a force is applied to the body, it will deflect. Clearly, the deflection and its direction will be different for different forces. It may be of interest to know the direction of the unit force which will cause the largest translation and that which will cause the smallest. Equivalently, the three available single DOF modes are presented (Figure 6). Then, a decision maker (subject 001-F) would interpret this by perceiving that the largest deflection direction as the most compliant direction [31] and the smallest deflection is the stiffest direction. The performer would take the most compliant direction ratio as an optimal policy and copy its information into his/or internal model.

**Figure 7:**
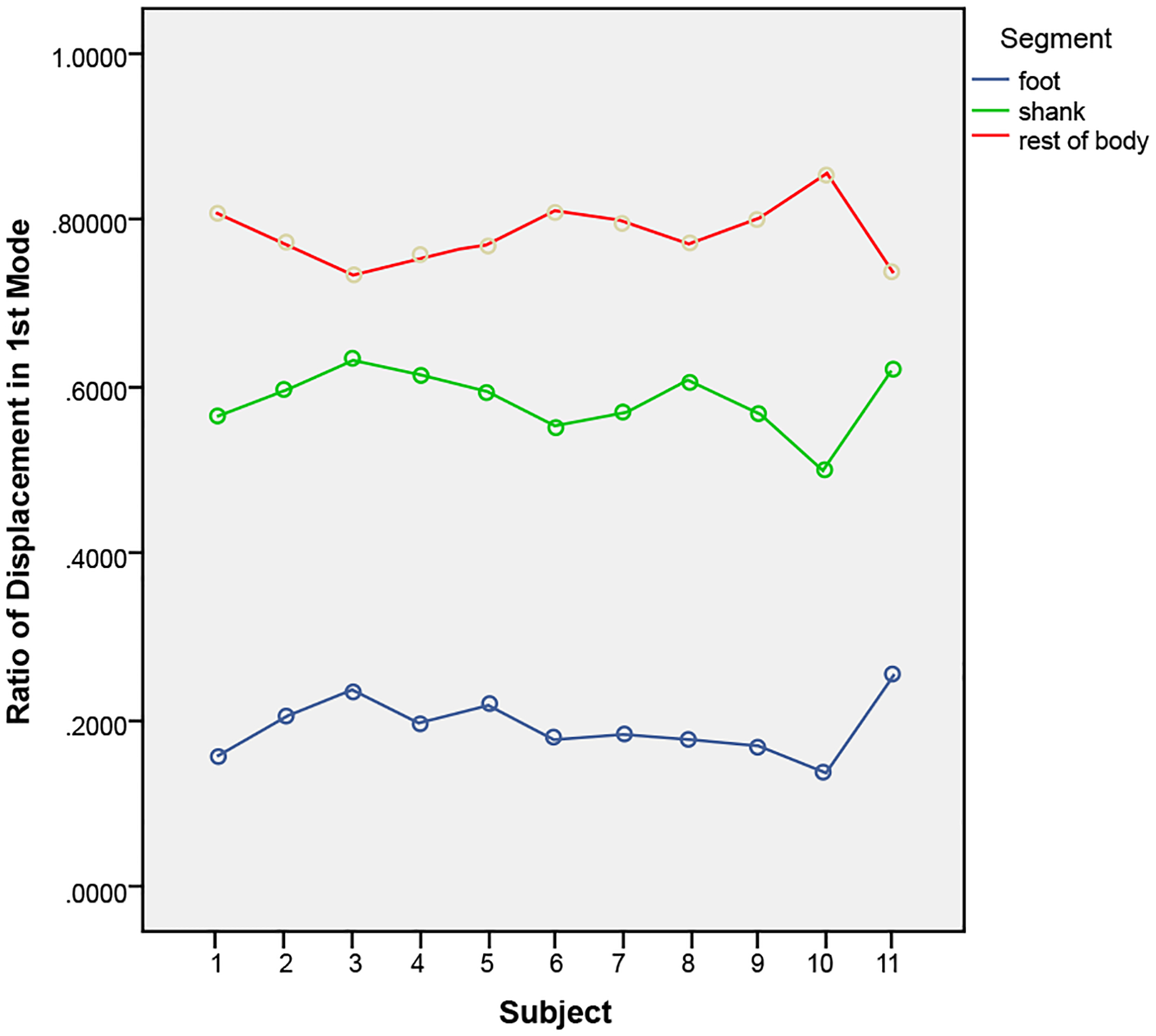
Summary of all subjects indicating unique mechanisms of stiffness adjustment within each individual’s leg spring to tune the foot-surface perception, permitting the vibration suppression function.

We have demonstrated that an efference copy that was generated in terms of three numbers as a specific ratio during the stance phase can be used to explain the interaction between performer and external environment. Therefore, the shape of the compliant direction in the efference copy can be regarded as the minimal unit of analysis of the hopping pattern under all load bearing physiologic conditions. Our investigation demonstrated that for the range of the studied performers, there existed statistically significant differences (p<0.01) in the ratio over subjects, showing subject specific patterns (Figure 7).

**Figure 8:**
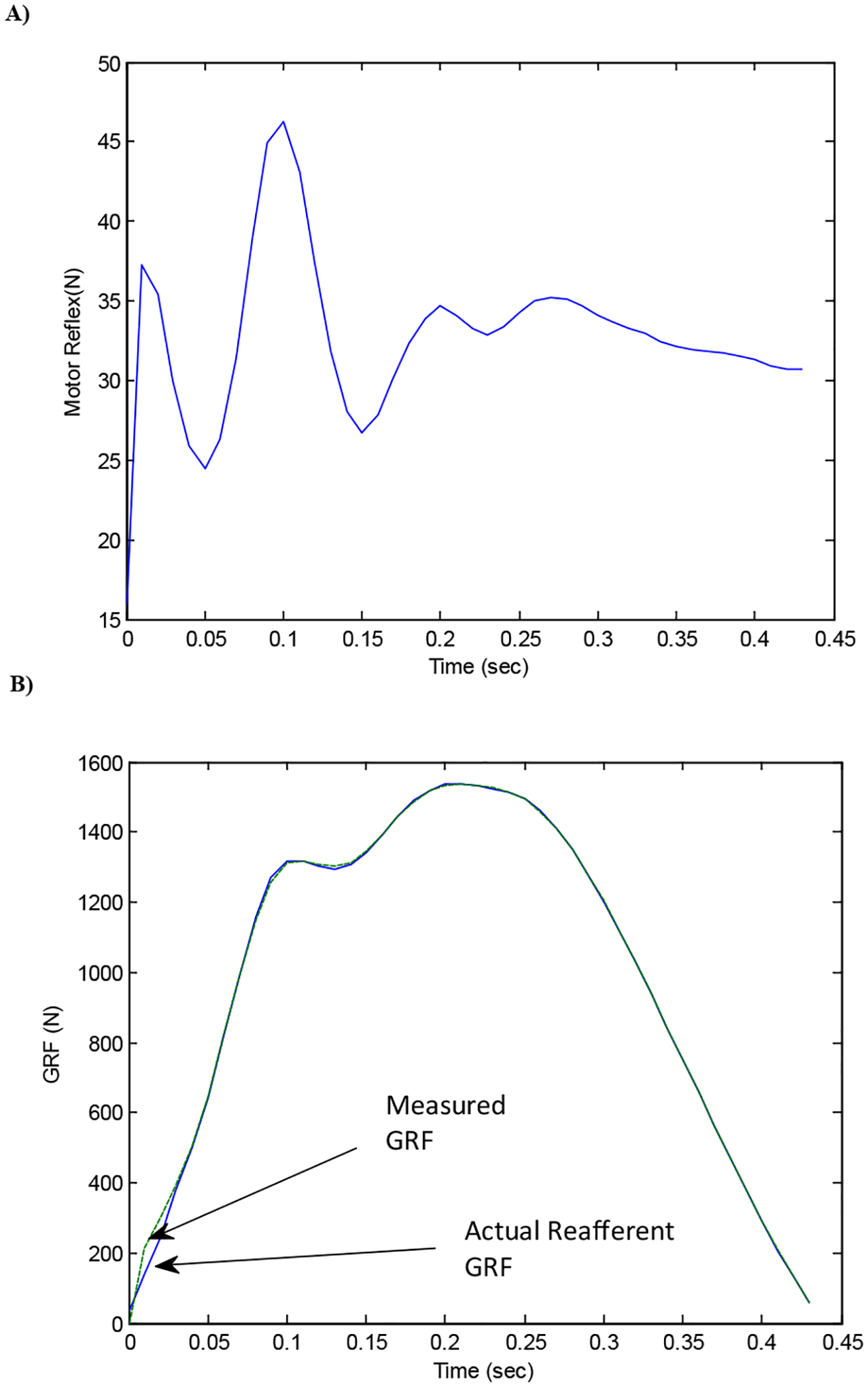
Representation of the internal model cancelling the reafferent signals. (A) The frequency of the estimated motor reflex was observed as 75.4 rad/sec, faster than the 6.5 rad/sec produced in the internal model. The internal model generated an estimate of reafference: the difference between this estimate and the actual reafferernt GRF can inform the motor reflex about external events. (B) When the motor reflexes at the foot-floor level were added to the reafference generated by the internal model, the actual reafference closely match with the measured GRF (Root Mean Square Error=6.5 N).

**Figure 9:**
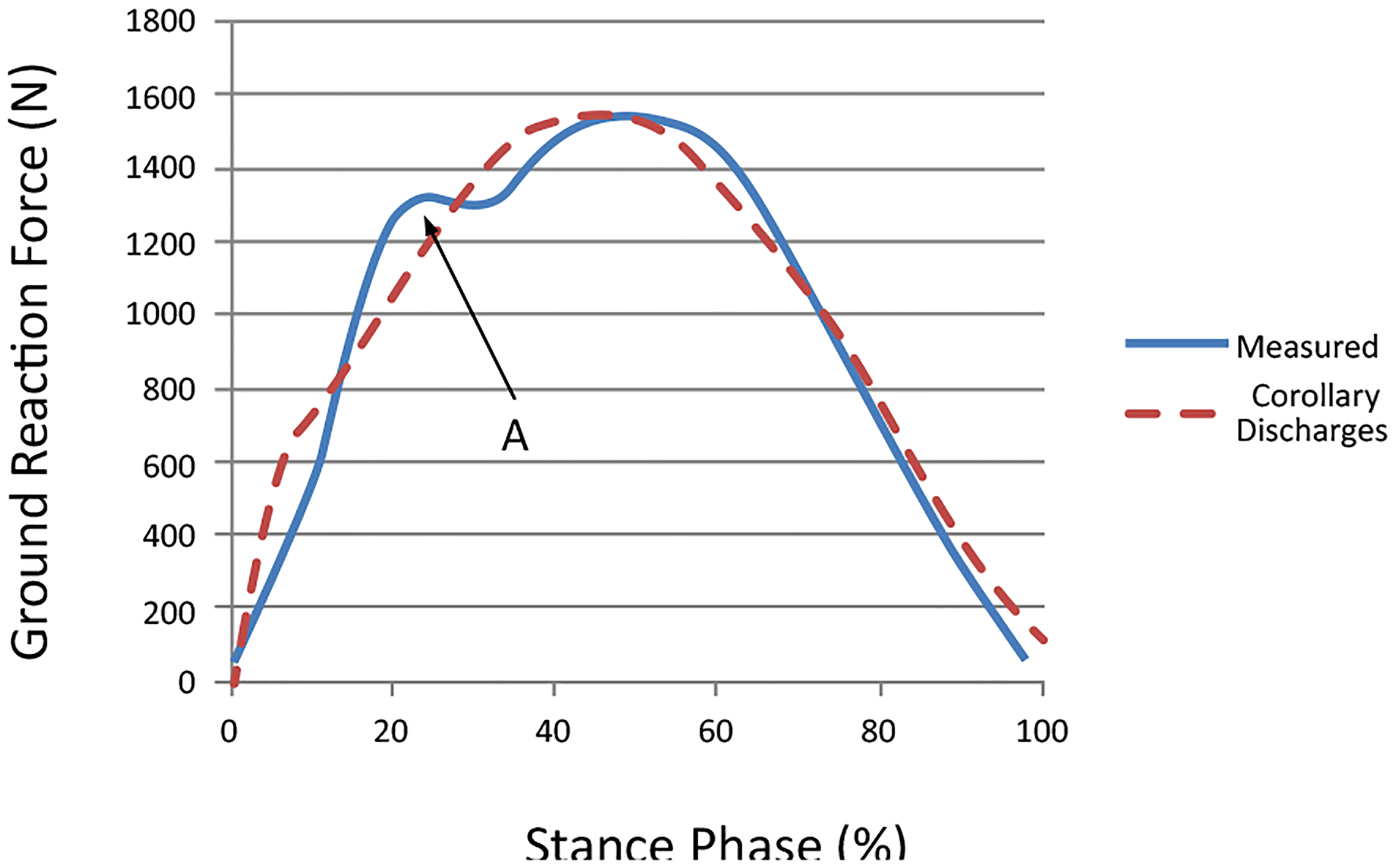
A plot of the isolated corollary discharge, which was limited by the fact that it has not reproduced the initial impact force with high frequency (indicated by A) in the estimated sensory feedback. Our efference copy assumed that the parameters defined as the leg spring were constant over the entire cycle of stance phase in the modeling process.

An important result from this investigation demonstrates an approach to separate ‘afferent’ sensory stimuli (Figure 8) from self-generated, ‘reafferent’ signals (Figure 9) using an action-oriented perception system and dynamic programming. The reflex action used to neutralize the disturbances between reafferent and afferent stimuli represents their difference.

**Figure 10:**
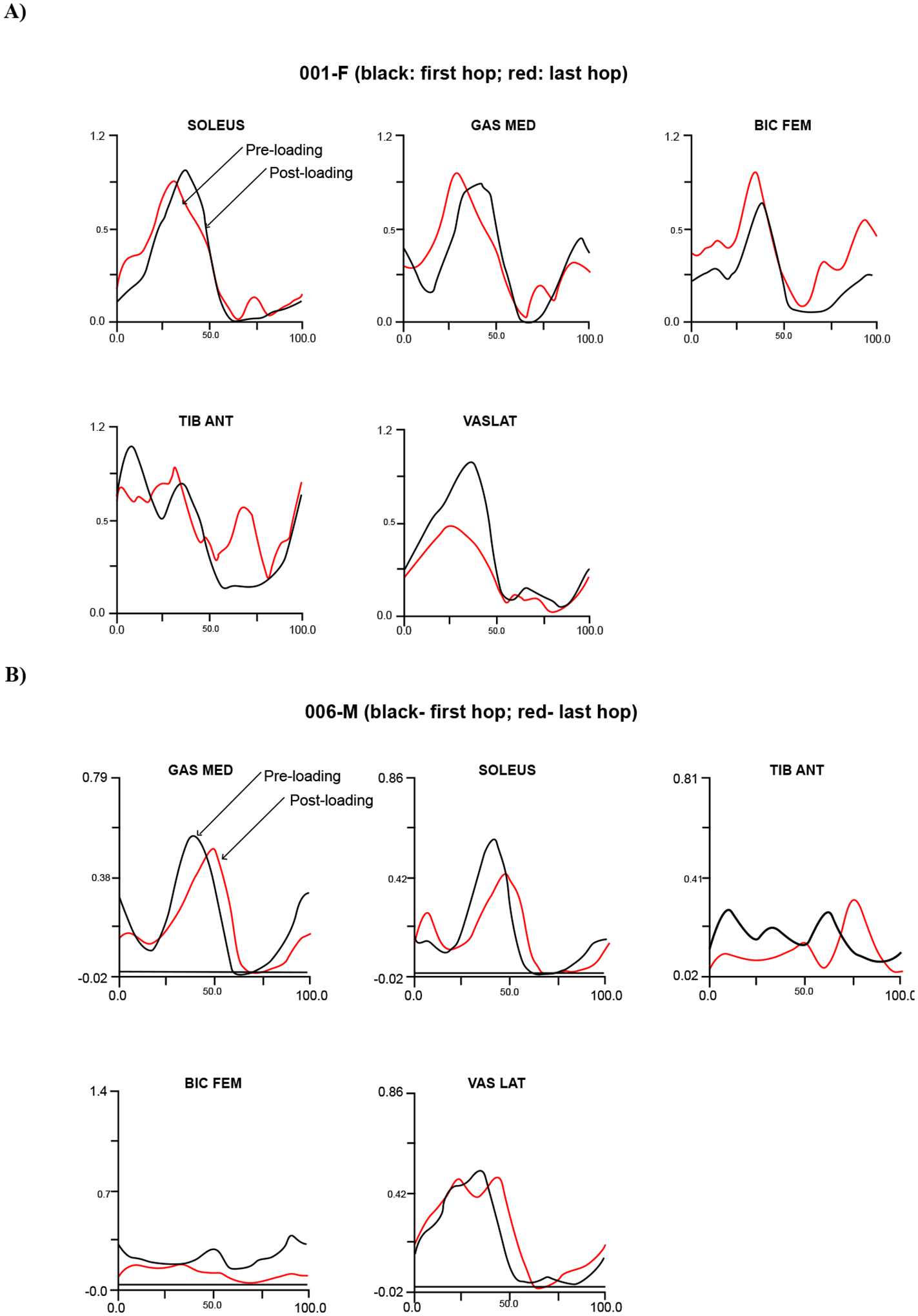
A comparison of muscle activation patterns through the recorded EMG signals acquired during the initial hop and the final hop activities. (A) The subject F-001 produced similar patterns in the involved muscles between post-loading at the first hop and pre-loading at the last hop during the stance phase. (B) The subject M-006 also produced similar patterns in the involved muscles with pre-loading at the first hop and post-loading at the last hop. The EMG signals were low-pass filtered using a fourth order Butterworth filter with a cut-off frequency of 6 Hz and normalized to peak activity recorded during the hop exercise.

**Figure 11:**
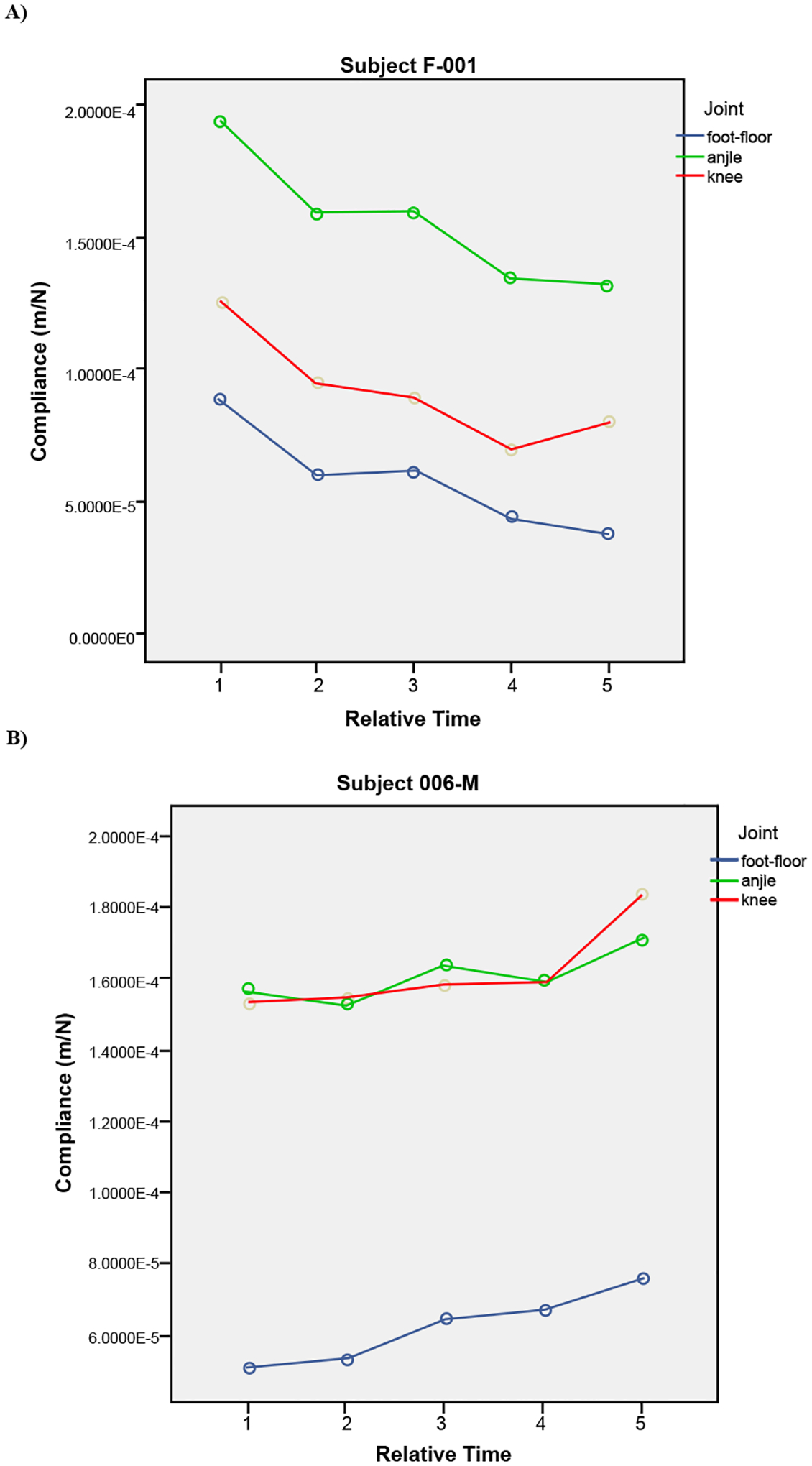
(A) The subject F-001 indicated a decrease in leg compliance during the hopping regime, as correlated with the EMG signals shown Figure 10A. (B) The subject M-006 indicated an increase in leg compliance, as correlated with the EMG signals in Figure 10B produced while nearing the fatigue condition.

EMG signals obtained from the five monitored muscles within two representative subjects (001-F and 006-M) represent the muscle activities during the stance phase of hopping and produced contrasting timing patterns (Figure 10). The two subjects also produced contrasting mechanisms of limb stiffness as physical adjustments during the five-hop regime (Figure 11).

## Discussion

We have demonstrated that when the leg spring chain has been displaced during the first harmonic mode, the forces in the leg spring no longer equilibrate. They create resultant force modes, intensities of which are decomposed into muscle tuning activities about the respective leg spring components [32]. Hence, if the equilibrium is stable during the stance phase, the evoked forces within the leg spring will tend to create motion for the leg to spring back to the position of equilibrium, and thus produce oscillation about the same harmonic mode. Our study addressed that the mechanism of stiffness adjustment is done via motor synergy about the leg joints. Generating the subject specific internal mode by motor synergy can be used as a possible strategy to affect the natural shock absorption ability during hopping.

Contrasting muscle activities were observed from two representative performers: namely pre-loading and post-loading (as indicated) muscle activity generated during the first and last hops (Figures 10 and 11). The subject 001-F produced the post-loading pattern at the first hop and the pre-loading at the last hop, along with increased stiffness in her leg spring; whereas, the subject 006-M produced the opposite pattern, along with decreased stiffness in his leg spring. The muscle activities that were observed at the first and last hop can be directly related to the mechanism of stiffness adjustment. We assume that the control forces within muscle activation simply added the new stiffness value to the existing leg spring system, which resulted in an increase in leg stiffness. In other words, the active control system with the control input is equivalent to the passive leg spring system with an increased stiffness (as illustrated in Figure 2).

The results may be explained in terms of synaptic enhancement through an increased probability of synaptic terminals releasing transmitters in response to pre-synaptic action potentials [33]. Such synaptic enhancement can, as modeled during repeated trials here, tend to keep the performers’ motor system on a customary path in a sensory feedback or corollary discharge system. Since memories are postulated to be represented by vastly interconnected networks of synapses in the brain, synaptic enhancement is one of the important neurochemical foundations of learning and memory [34]. Thus the efference copy reduces the order of the system where specific modes are selected as approximations. The selection of a small number of mechanical modes which approximate the assumed state variables are referred to as ‘Bernshtein’s reduction, of DOF [35].

The sensory system responds not only to stimuli from the environment but also cues generated by a performer’s own behavior. This leads to problems in sensory processing, because self-generated information can occur at the same time as external sensory information is gathered. However, this sensory information can also desensitize the performer’s own sensory pathways and they can become confused with external afferent information of the same modality. Although the afferent and reafferent signals have distinct causes, they are carried by the same neuronal channels. From a behavioral viewpoint it may be necessary to distinguish between signals originating from two causes especially when monitoring changes in the external environment separate from those resulting from self-movement [36]. Feed forward or corollary discharges are necessary to cancel reafferent inputs so that the self-generated, or reafferent sensory information is used to update or fine-tune the ongoing motor act. In an active sensory system this helps to monitor the environment.

The efference copy of hopping performers drives the motor activities and produces the corollary discharges that estimate sensory feed back of the GRF during the stance phase. However, an efference copy cannot itself provide this information, as it is a motor signal predictive of muscle activation, rather than of the sensory input (GRF). By generating an estimate of the sensory consequences of a motor command, an internal forward model can be used to cancel reafferent sensory signals, and thus allow the external environmentrelated signals to be recovered. Here, the self-generated GRF was compared to the measured GRF. The estimated sensory response, however, was limited by the fact that it did not reproduce the first spike (impact force) accurately in the simulation of the GRF data (Figure 9). Our work assumed that the parameters defined as the leg spring remain constant over the entire cycle of stance phase within the internal model. If additional motor components are identified, hoppers may respond not only to stimuli from the cues generated by their own behaviors but also to disturbances produced by the external surfaces. We call this phenomenon a ‘corollary discharge inhibition of the proprioceptive system in striding performers.’ As such, corollary discharge tends to influence an inhibition in the hoppers’ proprioceptive and cutaneous receptors during the hopping activity.

In this case the coordination of the motor components is ‘hardwired,’ consisting of fixed neuromuscular pathways as a reflex. There flex action is a brief stereotyped movement carried out in automatic fashion in response to some sensory stimulation. Performers avoid, escape from, or minimize the effects of noxious stimuli. It has also been perceived as a disturbance, which assumes the sensory discrepancy signals as a training model [37]. Differences between in the cutaneous receptors are evident here (Figures 8 and 9). This signal is regarded as a compensatory stabilizing reflex, which keeps the body fixed in space. These reflexes are regulated by sensory feedback from proprioceptors, which signal relative body segment motion. In addition, vestibular, visual, and tactile receptors signal head or body motion with respect to vertical position during hopping.

Reflexes are typically characterized as automatic and fixed motor responses, and they occur on a much faster time scale with a higher frequency than what is possible for reactions that depend on perceptual processing [38]. Reflexes play a fundamental role in stabilizing the motor system, providing almost immediate compensation for small perturbations and maintaining fixed execution patterns that are predefined by the efference copy. Thus they do not require attention or conscious control. The concept of the motor unit that was first introduced by Sherrington [39] can be divided into ‘fast twitch’ (policy in Figure 8) and ‘slow twitch’ (policy in Figure 9) response types.

We have demonstrated that performers continually need to make adjustments in order to maintain a predefined policy in the efference copy. These movements consist of a series of regulating reflexes aimed at restoring the body’s equilibrium, brought into play whenever the body deviates from its desired orientation. In addition to regulation responses, there is a second class of equilibrium responses, termed compensatory responses. These responses do not correct deviation from equilibrium; instead they compensate or neutralize the influences of the external noise. This compensation is not a feedback path, since the value of the input is transmitted along it and not the output variable of the object.

The rapid response mode may have some clinical implications as it directly affects the reflex proprioceptive system as a compensation mechanism. Therefore, we demonstrated that by separating self-generated motion from the external influenced motion, filtered information could be acquired for diagnosis. Recent research has identified neural pathways and their sensory processing as being highly dynamic, taking the behavioral state of the individual into account [40]. This indicates that the analysis of sensory pathways in anaesthetized or resting preparations might not provide the full picture of sensory processing. Therefore, our study suggests that future work should focus on unraveling the dynamics of sensory processing in active performers.

## Figures and Tables

**Table 1:** Parametric descriptors of the anatomic analogies associated with Figure 2.

Anatomic analogy	Leg parameters
Mass of the foot (e.g. 0.928 kg)	*m*_1_
Mass of the shank (e.g. 2.976 kg)	*m*_2_
Mass of the rest of the body (e.g. 51.648 kg)	*m*_3_
Motor synergy at the foot-floor contact including ankle flexor tendon, plantar fascia and the ligaments of the arch (N/m for spring k_i_ and Ns/m for dashpot c_i_)	*k*_1_ (*c*_1_)
Motor synergy of the bony contacts, ligaments and cartilage, and those involving muscle actions surrounding the ankle joint	*k*_2_ (*c*_2_)
Motor synergy at the knee joint	*k*_3_ (*c*_3_)
Position (or velocity) of *m*_1_	$x_{1}\left({\dot{x}}_{1}\right)$
Position (or velocity) of *m*_2_	$x_{2}\left({\dot{x}}_{2}\right)$
Position (or velocity) of *m*_3_	$x_{3}\left({\dot{x}}_{3}\right)$
